# Supplementary material for: Long-term cardiology outcomes in children after early treatment for Chagas disease, an observational study
Source: PLoS Negl Trop Dis. 2022 Dec 19;16(12):e0010968. doi: 10.1371/journal.pntd.0010968 (PMC9810160; doi:10.1371/journal.pntd.0010968)
Supplement: Attachment — Submitted filename: List of responses.docx [file pntd.0010968.s002.docx]

Buenos Aires, October 24th, 2022

**Ref. No.:** PNTD-D-22-00947

Dear Andrea Angheben and Ricardo Fujiwara,

I am glad to know that our manuscript is potentially acceptable for publication in PLOS Neglected Tropical Diseases. We appreciate the time that you and the reviewer spent looking over our work, and we believe that the comments and suggestions will improve the quality of the presented data.

After an exhaustive review of the Manuscript PNTD-D-22-00947, entitled “Long-term cardiology outcomes in children after early treatment for Chagas disease” we send the modifications following the suggestions issued hoping to be able to fulfil the expectations. Below you will find our own replies and comments to each point.

We look forward to hearing from you regarding our submission. We would be glad to respond to any further questions and comments that you may have.

**Methods**

Reviewer 1

The objectives of the study are clearly stated. They use cardiological, parasitological and serological assessments to analyze the final outcomes of the cohort of children diagnosed with CD.

The study design is the most appropriate to address the objectives. The population is clearly described.

**RESPONSE**

Thank you for your comments.

They also describe the median time of follow-up per patient. However, I missed the total follow-up time of the whole cohort.

Median follow up time was 9.8 years (6 - 20.2 years). For a better description IQR was included. (Line 183).

Additonally, when they are talking about diagnosis criteria for CD they do not include PCR as an option, mainly when later on the text (line 141) the serology and PCR are included to assess the parasitological results. If the endpoint is to determine the negativity of the PCR test for T cruzi, why do not include this test as diagnosis criteria for CD?

Diagnosis criteria was in infants younger than 8 months, CD was diagnosed by direct observation of *T. cruzi* using a parasitological concentration method (microhematocrit test - MH) or xenodiagnosis, and in infants older than 9 months by two reactive serological tests (ELISA, and indirect hemagglutination - IHA).

Following the standard diagnosis criteria (WHO).

PCR was not available in our service at the time of diagnosis in most of the patients. Then, PCR was not included as diagnostic criteria.

Regarding the analysis, I was wondering if the authors could add a table to compare outcomes between younger children (<12m-old) and the rest of the cohort for the serological, parasitological and cardiological results. I'm asking for this because the authors state that an early diagnosis is crucial for the final outcome regarding cardiological involvement.

Our aim was to describe the cardiological follow up of treated patients. We didn’t observe differences between age groups of treated patients.

In previous publications, we have described the serological profile of treated patients where we find differences between age groups. Younger patients showed an earlier seroreduction/seroconversion of *T.cruzi* antibodies.

Falk N, Berenstein AJ, Moscatelli G, Moroni S, González N, Ballering G, et al. Effectiveness of Nifurtimox in the Treatment of Chagas Disease: a Long-Term Retrospective Cohort Study in Children and Adults. Antimicrobial Agents and Chemotherapy. 2022 May 17;66(5).

Moscatelli G, Moroni S, García Bournissen F, González N, Ballering G, Schijman A, et al. Longitudinal follow up of serological response in children treated for Chagas disease. PLoS Neglected Tropical Diseases [Internet]. 2019 Aug 29 [cited 2022 Oct 14];13(8):e0007668. Available from: https://www.ncbi.nlm.nih.gov/pmc/articles/PMC6715178/

Ethics statement do not show a sentence regarding data protection regulation issues.

It was added as requested. (Line 170-171).

Reviewer 2

The results of this article provide extremely interesting data on a subgroup of patients on which there is not much published work as is the case of children. the article is well written and reflects the effort and work of a group of researchers of consolidated name.

The children were treated and clinically accompanied with a wide variety of serological techniques, molecular biology and complementary tests, which is undoubtedly of the highest scientific value.

**RESPONSE**

Thank you for your comment.

my only comment I am going to make about the article is that patients treated and accompanied for an average of 10 years are found practically no cardiological event. the authors attribute the absence of these events to the effect of the drug but in my opinion, there is no basis to think that it was the drug itself since there is no control group or comparator. the authors explain well that it would not be ethically accepted to have patients diagnosed with Chagas disease and not offer them treatment, but it would have been interesting to have had a cohort of patients without Chagas disease who had the same follow-up protocol.

We agree with your comment, but as we explained there are ethical issues that do not allow enrollment of an untreated group.

This is a limitation of the study to assign the absence of cardiologic events to treatment. However, this is the best approach we have found.

On the other hand, it would be very

clarifying to be able to understand based on which criteria the electrocardiographic alterations have been attributed or not to Chagas disease.

We used previous reports that describe the incidence of electrocardiographic alterations in patients with Chagas disease. In order to confirm this results, the patients continue in a prospective follow-up.

Reviewer 3

The study design is appropriate and the objectives clearly articulated

**RESPONSE**

Thank you for your comment.

**Results**

Reviewer 1

The results are clearly presented, with a good reading of all of them. As I said in the methods, I missed a table to show the results comaring younger children and the rest of the cohort, including a p-value (likely to be not statistically significant) in it.

Our aim was to describe the cardiological follow up of treated patients. We didn’t observe differences between age groups of treated patients.

In previous publications, we have described the serological profile of treated patients where we find differences between age groups. Younger patients showed an earlier seroreduction/seroconversion of *T. cruzi* antibodies.

Median follow-up time per patient is 9.8y, but what about the total follow-up time of the cohort? I would include this result in the first paragraph of this section.

The median age at diagnosis of CD is 62.5m with a range of 1 to 202 months (which IQR has the age at diagnosis?). This data is surprising for me because I had understood from previous CD studies that the best age for starting the treatment of CD should be when children are <12m-old.

For a better description IQR was included. (Line 182).

Have the authors observed any differences between thes age-groups?

No differences were observed between children younger or older than 12 months old.

I was wondering about the 3 cases (table 1) with abnormal ECG findings that were not considered related to CD, why do you not consider these findings related to CD? Case 1 was diagnosed at 3y and the evaluation was performed at 19y. This case serorreverted (ELISA result is neg) but you considered that cardiological findings were related to CD, on the contrary other cases with positive serological results had cardiological findings that were not considered related to CD, could you better explain these issues to the readers? Thank you.

We used previous reports that describe the incidence of electrocardiographic alterations in patients with Chagas disease. In order to confirm these results, the patients continue in a prospective follow-up.

Regarding the patient with RBBB, it is an alteration that was described in the literature related to ChD. It justified that we assign this alteration to ChD. However, there are other causes that could justify its appearance.

Any age-related outcomes? I would add a table showing the differences.

Has the age at diagnosis an impact for the final parasitological or serological or cardiological outcomes?

In previous publications, we have described the serological profile of treated patients where we find differences between age groups. Younger patients showed an earlier seroreduction/seroconversion of *T. cruzi* antibodies.

In line 221 the same sentence is repeated twice.

Thank you for your comment. It was modified as suggested.

Reviewer 2

see above

-

Reviewer 3

Overall they found ECG abnormalities in 4.7% of subjects (11/234) but according to their interpretation only 2/234 (0.8%) were related to Chagas disease. Although I’m not a cardiologist I’m not sure that “Left anterior fascicular block (LAFB)” observed in two subjects (both 16 years-old) can be regarded as “normal pediatric ECG abnormalities” and not as CD- related alterations. LAFB is commonly considered an early ECG sign of Chagas cardiomyopathy, both subjects still have an ELISA reactive test and probably as for their age should be considered adults. Are the authors able to produce more convincing evidence that such patients should be dismissed as not having CD-alterations?

It was modified in the manuscript. (Line 41, 188, 189, 203, figure 1).

In table 1 could be useful add a column reporting other cardiological evaluation (i.e. Holter, echo, STE) if such exams were done.

It was added. (Table 1).

Figure 1 is unclear how many of the 11 patients with ECG alterations underwent Holter or STE.

This information is only found in the text (7/11 patients had Holter monitoring), as it was not our intention to continue adding more information in the figure. We modified the manuscript, and now it is clearer in the text. (Line 201).

Regarding treatment can you report how many patients completed the scheduled therapy in both groups?

It was added. (Line 227).

Page 15 line 221-222 the sentence is reported twice.

It was modified.

Page 15 line 224-225: “A decrease in T. cruzi antibody titers by conventional serology (IHA and ELISA) was observed after treatment. This is not reported and I think you should provide such data.

See figure 2.

**Conclusions**

Reviewer 1

The conclusions are supported by the data presented.

Thank you for your comment.

However, in line 244 the authors comment on early treatment but the median age at diagnosis for the cohort was not as early as recommended by international health institutions such as the WHO.

The supporting information sheet is empty and I cannot evaluate it.

Early treatment refers to the administration of treatment as soon as possible in children, not only in patients younger than 12 months old.

The public health relevance is clearly addressed by the authors.

Thank you for your comment.

Reviewer 2

see above

-

Reviewer 3

Yes the conclusions are supported by the data presented but the number of patients with cardiological alterations should be changed (see my comments on the results)

It was modified in the manuscript.

Only 41% subjects underwent Holter and STE; this should be reported among the study limits

It was added as requested.

**Editorial and Data Presentation Modifications?**

Reviewer 1

(No Response)

-

Reviewer 2

(No Response)

-

Reviewer 3

Some references are outdated or inappropriate (for instance ref. 2,3,4,5). Please consider the following:

Bern C et al. Chagas disease in the United States: a public health approach. Clin Microbiol Rev 2019;23(1):e00023-19.

Abras A et al. Worldwide control and management of Chagas disease in a new era of globalization: a close look at congenital Trypanosoma cruzi infection. Clin Microbiol Rev 2022; 35(2):e0015221.

Imai K, et al. Chagas disease : a report of 17 suspected cases in Japan, 2012-2017. Trop Med Health 2019;47:138.

Freitas Lindani KC et al. Chagas disease from discovery to a world wide health problem. Front Public Health 2019;7:166.

It was modified.

**Summary and General Comments**

Reviewer 1

(No Response)

-

Reviewer 2

In summary, paper with a very high scientific value on a population of which we are not used to having data but the authors would have to minimize or at least contextualize the effect of the drug on the clinical evolution of patients

We said it in the manuscript:

-“It is likely that this was related to the protective effect of treatment at an early stage of *T. cruzi* infection.”

-“Further studies with long-term follow-up (20-30 years) of the present cohort will be conducted in order to confirm that early treatment prevents later cardiological compromise in CD patients.”

Reviewer 3

This is an interesting study conducted in Buenos Aires (Argentina) in a cohort of children aiming to evaluate the cardiological outcome post-treatment for Chagas disease with a median follow-up of 9.8 years.

As reported by the authors this issue has been previously evaluated in a limited number of studies some of which biased by the lack of appropriate ECG interpretation by a pediatric cardiologist.

Thank you for your comment.

Only 41% subjects underwent Holter and STE; this should be reported among the study limits

It was added. We reported in the manuscript about the study limits.

We believe that we have addressed the reviewers’ comments in this revised version and are sending it to you for further consideration for publication in PLOS Neglected Tropical Diseases. Thank you for your time and we look forward to hearing from you soon.

Yours sincerely,

Nicolás González

Jaime Altcheh

Servicio de Parasitologia, Hospital de Niños Ricardo Gutiérrez, Buenos Aires, Argentina

Gallo 1330, 1425 Buenos Aires, Argentina

Email: ng211@hotmail.com

Phone/Fax: (+5411) 4963-4122.
